# Antagonistic Regulation of ABA and GA in Metabolism and Signaling Pathways

**DOI:** 10.3389/fpls.2018.00251

**Published:** 2018-02-26

**Authors:** Xu Liu, Xingliang Hou

**Affiliations:** Key Laboratory of South China Agricultural Plant Molecular Analysis and Genetic Improvement and Guangdong Provincial Key Laboratory of Applied Botany, South China Botanical Garden, Chinese Academy of Sciences, Guangzhou, China

**Keywords:** gibberellic acid, abscisic acid, antagonistic interaction, metabolic pathway, signaling pathway

## Abstract

The phytohormones gibberellic acid (GA) and abscisic acid (ABA) are widely recognized as essential endogenous regulators that mostly play antagonistic roles in plant developmental processes and environmental responses. A variety of both internal and external cues oppositely regulate GA and ABA biosynthesis and catabolism, which directly and indirectly affect their signaling pathways and subsequent responses. Recent discoveries have revealed direct molecular links between GA- and ABA-signaling components, which provide novel insights into their antagonistic regulation. In this review, we mainly focus on these recent reports and the growing understanding of GA and ABA antagonism in metabolic regulation and signaling interactions, and attempt to clarify the problems and challenges involved in exploring the complicated regulatory events associated with these two phytohormones.

## Introduction

In higher plants, the classic phytohormones abscisic acid (ABA) and gibberellic acid (GA) antagonistically regulate various developmental stages, such as seed dormancy and germination, root growth, leaf development, flowering time, and responses to environmental cues, such as light, temperature, and abiotic stresses (Weiss and Ori, 2007; Vanstraelen and Benková, 2012; Golldack et al., 2013).

Gibberellic acid form a large class of diterpenoid compounds in plants and fungi, but only a handful of GAs are bioactive and essential for plant growth and development (Yamaguchi, 2008). In GA biosynthesis and catabolism, various non-bioactive GAs act as precursors and intermediates in the conversion to bioactive GAs, which are mainly catalyzed by GA 20-oxidases (GA20ox) and GA 3-oxidases (GA3ox). Otherwise, the existing bioactive GAs are deactivated to non-bioactive forms, which are catalyzed by GA 2-oxidases (GA2ox) or another P450 monooxygenase (e.g., elongated uppermost internode) in Arabidopsis or rice (Sun, 2011). Thus, GA20ox and GA3ox are key synthetic enzymes of bioactive GAs and positively regulate the GA metabolism, while GA2ox and ELONGATED UPPERMOST INTERNODE are crucial catabolic enzymes of bioactive GAs and negatively regulate GA metabolism.

The sesquiterpene metabolite ABA plays crucial roles in plant development as well as in adaptation to abiotic stresses (Nambara and Marion-Poll, 2005). Cellular ABA biosynthesis occurs mainly in chloroplasts and cytoplasm. First, the carotenoid zeaxanthin is catalyzed to the all-*trans* violaxanthin in the chloroplast by zeaxanthin epoxidase (ZEP). Second, the intermediate violaxanthin is catalyzed to xanthoxin by nine-*cis* epoxycarotenoid dioxygenase (NCED), which is transported from the chloroplast to the cytoplasm. Then, xanthoxin is converted to abscisic aldehyde and oxidized to bioactive ABA by short-chain dehydrogenase reductase and abscisic aldehyde oxidase in the cytoplasm (Seo and Koshiba, 2002). The bioactive ABA is catalyzed to hydroxy ABA (ABA deactivation) by cytochrome P450 monooxygenase (CYP707A) superfamily members (Nambara and Marion-Poll, 2005). Thus, the key ABA synthetic enzymes are ZEP, NCED, short-chain dehydrogenase reductase, and abscisic aldehyde oxidase, which positively regulate ABA metabolism, while CYP707As negatively regulate ABA metabolism by catalyzing the ABA deactivation reaction.

In the current model of GA action that has been well-identified in rice and Arabidopsis, DELLA proteins interact with key regulatory factors, transcriptionally regulating downstream genes to restrain plant growth and development, while GA promotes growth by releasing DELLA-inhibition to activate the GA response (Davière and Achard, 2013). Similarly, ABA mediates its signaling pathway through a double-negative regulatory system. PP2C represses the positive ABA regulator SnRK2 activity, while ABA recognizes PP2C via receptor to releases activated SnRK2 that phosphorylates downstream targets, such as ABA RESPONSIVE ELEMENT BINDING FACTOR proteins (ABFs), to activate the ABA response (Cutler et al., 2010).

The essential phytohormones GA and ABA play diverse and contrary roles in plant growth and development processes at the physiological, biochemical, and molecular levels. However, the molecular mechanisms behind the antagonistic actions of these two hormones are not fully understood and have only been investigated using elusive and complicated regulatory networks. Here, we review recent advances in two aspects of GA and ABA antagonistic interactions, metabolic regulation, and core molecular signaling.

## The Antagonistic Regulation of GA and ABA Metabolic Genes in Plant Development and Stress Responses

The antagonistic regulation of metabolic genes is a main feature of the GA and ABA interactions, and these interactions have mostly negative correlations at different developmental stages, including light-mediated seed germination, seed maturation and dormancy, root growth, and in response to stresses, such as cold, high temperature, and high salt.

Studies of the photoreversible seed germination of Arabidopsis revealed the opposing regulatory mechanisms of GA and ABA metabolism. For instance, bioactive GA biosynthesis is up-regulated by the light-mediated phytochrome pathway, which mainly activates the transcription of GA biosynthetic genes *GA3ox1* and *GA3ox2* and represses the transcription of the GA deactivation gene *GA2ox2* to promote active GA accumulation in the light-regulated seed germination process (Yamaguchi et al., 1998; Oh et al., 2007). At the same time, ABA biosynthesis and inactivation are also phytochrome-regulated by down-regulating the ABA biosynthesis gene *NCED6* and activating the ABA deactivation gene *CYP707A2* in a manner opposite to that of GA metabolism (Seo et al., 2006). More importantly, high GA levels in the ABA-deficient mutants are observed, indicating that ABA is involved in the suppression of GA metabolic genes (*GA3ox1*/*2* and *GA3ox1*/*2*/3) in seed germination, while the inhibition of GA elevates ABA metabolism by promoting the ABA biosynthetic genes (*ABA1*, *NCED6*, and *NCED9*) and repressing the ABA catabolic gene (*CYP707A2*) in the GA-deficient mutant seeds (Seo et al., 2006; Oh et al., 2007). Many recent studies focused on the regulation of GA and ABA metabolism to illuminate the potential regulatory mechanism involved in light-mediated seed germination. A light-mediated basic helix-loop-helix leucine zipper domain containing transcription factor (TF) in Arabidopsis, PHYTOCHROME-INTERACTING FACTOR3-LIKE5 (PIL5), inhibits GA metabolism in seed germination by indirectly repressing the GA synthetic genes *GA3ox1* and *GA3ox2* and activating a GA catabolic gene *GA2ox2*, and it increases ABA levels by indirectly activating the ABA synthetic genes *ABA1*, *NCED6*, and *NCED9* and repressing an ABA catabolic gene *CYP707A2* (Oh et al., 2007). In addition, the zinc finger protein DOF AFFECTING GERMINATION1, functioning downstream of PIL5, directly down-regulates the GA biosynthetic gene *GA3ox1* in light-dependent seed germination (Gabriele et al., 2010). Similarly, Arabidopsis SOMNUS/Tandem CCCH zinc finger 4 (SOM/TZF4) and its homologs TZF5 and TZF6 modulate the expression of ABA and GA metabolic genes and function in the light-, GA-, and ABA-mediated regulation of seed germination responses (Kim et al., 2008; Bogamuwa and Jang, 2013). The expression changes in ABA and GA metabolic genes in the *som/tzf4* mutant lead to lower ABA levels and elevated GA levels. Meantime, SOM is directly downstream of PIL5, which regulates ABA and GA metabolic genes partly through SOM (Kim et al., 2008). Moreover, CHOTTO1, a double-AP2 domain-containing TF, represses the GA biosynthetic gene *GA3ox1* and activates *ZEP* and *NCED9* genes to regulate ABA and GA metabolism during Arabidopsis seed germination (Yano et al., 2009).

In addition to light-mediated seed germination, another study on temperature-controlled seed dormancy clarified the temperature-dependent expression patterns of GA and ABA metabolic genes in the seasonal seed dormancy of Arabidopsis (Footitt et al., 2011). Increased dormancy by low temperature in the winter is correlated with the up-regulated expression of ABA synthesis (*NCED6*) and GA catabolism (*GA2ox2*) genes. Conversely, decreased dormancy by high temperature in the spring and summer is accompanied with increased ABA catabolism (*CYP707A2*) and GA synthesis (*GA3ox1*) (Footitt et al., 2011). In a study of the regulatory mechanism, ABA-INSENSITIVE 4 (ABI4), an AP2 domain-containing TF, was reported as the potential mediator between ABA and GA metabolism to promote seed dormancy by directly repressing the ABA inactivation genes *CYP707A1* and *CYP707A2* and inhibiting GA metabolism genes in Arabidopsis (Shu et al., 2013). The MYB96 TF regulates seed dormancy through ABA and GA metabolism by directly activating the ABA biosynthetic genes *NCED2* and *NCED6* and also repressing GA biosynthetic genes *GA3ox1* and *GA20ox1* (Lee et al., 2015).

In seed development, seed morphogenesis (embryogenesis) and seed maturation are considered two major physiological processes. A high level of ABA and low level of GA have been observed during seed maturation processes in some published studies (Hays et al., 2001; Yamaguchi et al., 2007), however, the GA and ABA levels during embryogenesis in developing seeds have been less reported. Embryo development and seed maturation are controlled by the B3 domain containing the TFs, LEAFY COTYLEDON2 and FUSCA3 (FUS3) in Arabidopsis, which antagonistically modulate ABA and GA metabolism by directly repressing the GA synthetic gene *GA3ox2* and promoting ABA synthesis through light-dependent DELLA stimulation and ABI3 activity (Curaba et al., 2004; Gazzarrini et al., 2004; Piskurewicz et al., 2009). Similarly, the B3 TF GERMINATION DEFECTIVE 1 directly or indirectly modulates GA homeostasis by suppressing rice (*Oryza sativa*) *LEAFY COTYLEDON2/FUS3-LIKE 1* and then regulates GA metabolic gene (*OsGA3ox*, *OsGA20ox*, and *OsGA2ox*) expression involved in seed maturation, germination, and seedling development (Guo et al., 2013).

The GA–ABA antagonism not only dominates seed development, dormancy, and germination, but also contributes to other developmental processes, which are also mostly promoted or suppressed by an opposite and specific TF-regulated ABA/GA ratio. The GA- AND ABA-RESPONSIVE ZINC FINGER factor modulates ABA and GA metabolism in Arabidopsis root tissue-formation by regulating *CYP707A1*, *GA20ox2/3/4/5*, and *GA3ox1/3/4* (Lee et al., 2016). ABI4, of which protein stability is oppositely regulated by GA and ABA, directly activates *NCED6* and *GA2ox7* transcription, suggesting the important role of ABI4 in ABA and GA antagonism during post-germination stages (Shu et al., 2016). In addition, OsAP2-39, may balance ABA and GA metabolism in multiple physiological processes, such as the size of root systems, flowering time, and pollen grain morphology, by directly regulating ABA and GA biosynthetic or inactivation genes (Yaish et al., 2010). Notably, both ABI4 and OsAP2-39 belong to the AP2 domain-containing TFs (ATFs), implying ATFs play key roles in the metabolism regulation of ABA and GA, which has been summarized recently (Shu et al., 2018).

Abiotic stresses (i.e., high temperature, cold, and high salt) trigger the relevant stress responses by affecting the balance of ABA and GA contents. High temperature increases the ABA level and decreases the GA level in Arabidopsis seeds (Toh et al., 2008). The increasing ABA level in imbibed seeds contributes to the up-regulation of the ABA synthetic enzyme genes *ZEP*, *NCED2*, *NCED5*, and *NCED9* at high temperatures. However, the decrease in the GA level at high temperature is largely the result of the suppression of the GA synthetic enzyme genes *GA20ox1*, *GA20ox2*, *GA20ox3*, *GA3ox1*, and *GA3ox2* (Toh et al., 2008). The Arabidopsis seed development master regulator FUS3 activates heat-related and ABA metabolic genes and inhibits the GA metabolic genes to delay germination at high temperatures (Chiu et al., 2012). Similarly, the DELLA proteins GAI or RGA, ABI3 and ABI5 directly activate SOM, which regulates ABA and GA metabolic genes during the high-temperature response in Arabidopsis (Lim et al., 2013). In addition, cold and high-salt stresses also result in changes in the ABA/GA ratio (Golldack et al., 2013). Low temperature- and ABA-induced C-REPEAT/DROUGHT-RESPONSIVE ELEMENT BINDING FACTOR 1 enhances the accumulation of RGA by inhibiting GA metabolism by promoting the expression of the GA catabolic gene *GA2ox* (Achard et al., 2008). The Arabidopsis AP2 domain-containing TF DWARF AND DELAYED FLOWERING 1 directly promotes the GA-inactivated gene *GA2ox7* to repress GA metabolism during high-salinity stress in Arabidopsis (Magome et al., 2008).

Thus, we can reasonably infer that the antagonistic regulation of GA and ABA metabolism mainly occurs by activating and repressing the opposing metabolic genes (*NCED*/*GA2ox* or *CYP707A*/*GA3ox* family) to maintain a hormonal balance during plant growth and development and to respond to environmental cues. The pattern information of phytohormone distribution could be acquired by recent data and tools, which will help us to gain novel insights into ABA and GA action in a temporal and spatial view (Waadt et al., 2015).

## The Antagonistic Interaction Between Core GA- and ABA-Signaling Components

Our knowledge regarding ABA- and GA-signaling pathways has substantially improved over the past two decades, although direct molecular interactions between core ABA- and GA-signaling components remains to be elucidated. Other phytohormone signals have been well-demonstrated and provide good references for the investigation of the direct interactions. For example, JASMONIC ACID ZIM proteins-DELLAs interaction for crosstalk between JA and GA (Hou et al., 2010; Yang et al., 2012), BRASSINAZOLE RESISTANT 1-DELLAs interaction for crosstalk between brassinosteroids (BR) and GA (Li et al., 2012), BRASSINOSTEROID INSENSITIVE 2-ABI5 interaction for crosstalk between BR and ABA (Hu and Yu, 2014), ARABIDOPSIS RESPONSE REGULATOR proteins-DELLAs for crosstalk between cytokinin and GA (Marín-de la Rosa et al., 2015), and DWARF 14-DELLAs for crosstalk between strigolactones and GA (Nakamura et al., 2013). Encouragingly, some novel interpretations may have revealed the direct molecular links between ABA and GA signaling.

The antagonistic GA–ABA signaling and downstream transcriptional regulations have been well-illustrated in barley aleurone cells. The GA- and ABA-mediated downstream genes encoding α-amylases are induced by GA but regulated by ABA in an opposite manner, and the key TF GAMYB is essential for activating α-amylase expression and is itself regulated positively by GA and negatively by ABA through core GA- and ABA-signaling components DELLA and PKABA1 (SnRK2 homolog), respectively (Gomez-Cadenas et al., 2001; Kaneko et al., 2004). However, the downstream targets of the antagonistic GA–ABA signaling are still not clear in the model plant Arabidopsis owing to the missing analogous α-amylase gene and relevant GAMYB TF.

In the core GA-mediated signaling, the DELLA repressors are mainly degraded through the ubiquitin–proteasome system. SLY1 recruits the Skp, Cullin, and F-box E3 ubiquitin ligase to the GA-GID1-DELLA complex and causes DELLA polyubiquitination and degradation in the 26S proteasome system (Steber et al., 1998; Murase et al., 2008). In addition to ubiquitination, the core signaling components are regulated by ubiquitin-like modifiers, such as small ubiquitin-related modifier (SUMO). Recently, the E3 SUMO ligase SIZ1 was found to negatively regulate ABA signaling through the sumoylation of ABI5, a core component of ABA signaling during seed germination in Arabidopsis (Miura et al., 2009). Interestingly, SIZ1 directly and positively regulates a GA-signaling component by sumoylating SLY1 (Kim et al., 2015), implying that SIZ1 functions antagonistically as a direct molecular link between SLY1 promotion and ABI5 inhibition in ABA and GA signaling.

ANAPHASE-PROMOTING COMPLEX/CYCLOSOME (APC/C) is another E3 ligase complex in the E3 family of enzymes and is necessary for many cellular events. Recently, the SnRK2-APC/C Tiller Enhancer (TE) regulatory module revealed a novel regulatory link underlying the antagonistic action of GA and ABA in rice. In this model, ABA inhibits APC/C^TE^ activity by phosphorylating TE by activating the OsSnRK2s, which may break the interaction between TE and the OsPYLs and subsequently stabilize the ABA receptor. In contrast, GA inhibits the OsSnRK2s and may promote the APC/C^TE^-mediated degradation of the OsPYLs (Lin et al., 2015).

DELLAs form a central connection between GA and other signaling pathways, including ABA signaling. The ABA-promoted accumulation of DELLA proteins in roots depends on ABI1, a core ABA-signaling repressor, and the phenotype of a quadruple-DELLA mutant resembles that of the gain-of-function mutant *abi1-1*, being resistant to the growth-inhibitory effects of ABA (Achard et al., 2006; Guo et al., 2014). By contrast, ABI1 does not mediate DELLAs’ stability induced by salt stress (Duan et al., 2013), suggesting that the regulation of ABI on DELLA proteins occurs specifically upon exogenous ABA applications. Similarly, the tomato DELLA protein PROCERA promotes stomatal closure in guard cells in an ABA-dependent manner (Nir et al., 2017). In transcriptional regulation, DELLAs regulate a set of downstream genes by interacting with the TFs involved in ABA signaling. For example, the Arabidopsis GA-signaling repressors GAI and RGA function in response to heat stress by interacting with core ABA-signaling TFs ABI3 and ABI5, which directly activate *SOM* expression and integrate ABA and GA signaling (Lim et al., 2013). In addition, Arabidopsis NUCLEAR FACTOR-Y C homologs (NF-YC3, 4, and 9) also interact with the core GA signaling repressor RGA-LIKE2 (RGL2) to form NF-YC-RGL2 modules that target the key ABA-signaling TF gene *ABI5* to regulate a set of GA- and ABA-responsive genes during seed germination (Liu et al., 2016). Interestingly, a recent study showed that NF-YC9 promotes ABA responses in early seedling growth and stomatal closure by binding ABI5 to increase ABA sensitivity (Bi et al., 2017), which further supports the hypothesis that the NF-YCs–DELLAs–ABFs/ABI5 module integrates the antagonistic GA and ABA signaling in seed germination and post-germination stages.

## Conclusion and Perspectives

**FIGURE 1:**
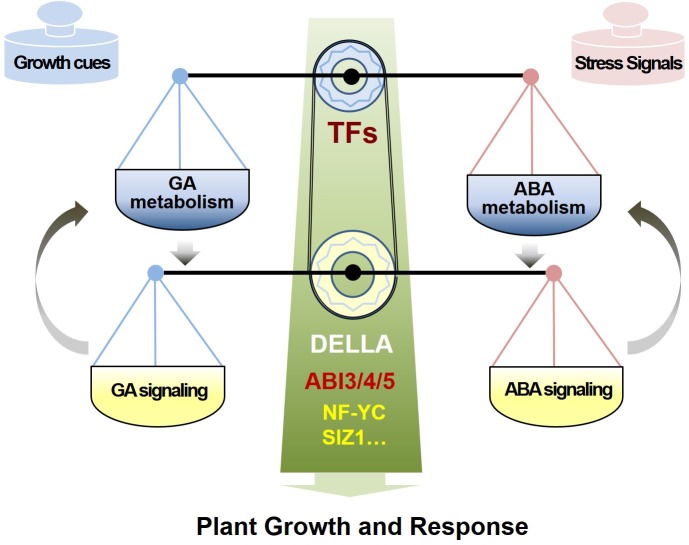
The model of antagonism of ABA and GA in metabolism regulation and signaling interaction. Phytohormones GA and ABA antagonistically mediate plant normal growth and response by two regulation layers, including the metabolic homeostasis of ABA and GA controlled by TF regulators in response to various environmental signals, and the direct molecular interaction between ABA and GA signaling components or other mediators, which orchestrate an efficient response to the cues of growth and stress.

To summarize, the plant hormones ABA and GA antagonistically mediate multiple physiological processes, and their balance is critical to normal development and stress responses. The GA and ABA antagonistic crosstalk comprises two main layers (**Figure 1**): one is the metabolic homeostasis of ABA and GA, which are controlled by distinct TF regulators in response to specific endogenous and environmental signals, leading to opposite patterns of ABA and GA accumulation; and the other one is the direct molecular interaction between core ABA- and GA- signaling components, which orchestrates a rapid and efficient response to developmental changes and external challenges by quickly mediating the antagonistic interaction of ABA and GA.

In recent years, many studies have shed light on the antagonistic regulation of GA and ABA in plants; however, the intricate regulatory network involved has made it difficult to develop a clear picture of plant growth and development as mediated by crosstalk between GA and ABA. Several notable points are worthy of further investigations: first, phytohormones relay signals through signaling pathways, while signaling components have feedback effects on phytohormone metabolism, which contributes to a beneficial hormone homeostasis balance for plant normal growth. Thus, how does the antagonistic regulation of metabolic and signaling pathways between GA and ABA synergistically control plant growth and development? Second, as the essential growth regulators, GA and ABA perform separate roles in different cells and tissues during various developmental stages. How is the GA–ABA antagonism precisely modulated in an accurate temporal and spatial manner to process a specific development such as germination or dormancy? Third, several epigenetic regulators, such as BRAHMA, HDAs, and REF6, have been shown to be involved in GA or ABA response (Hou et al., 2014; Ryu et al., 2014; Peirats-Llobet et al., 2016). Significantly, the GA–ABA antagonistic mediator NF-YC has a functional interaction with the epigenetic factors HDA15 or REF6 (Hou et al., 2014; Tang et al., 2017), implying that NF-YC may mediate GA–ABA antagonism by epigenetic regulation. In addition, some studies reported that non-coding RNAs, which act the overlapping or distinct functions in various molecular events, especially transcription regulation, are also related to GA or ABA response (Yu et al., 2012; Yan et al., 2016; Huang et al., 2017; Qin et al., 2017). Thus, it is worthy to investigate whether and how epigenetic factors and non-coding RNAs play roles in the crosstalk between GA and ABA.

## Author Contributions

XL and XH wrote the manuscript; XL and XH contributed to the discussion and approved the final manuscript.

## Conflict of Interest Statement

The authors declare that the research was conducted in the absence of any commercial or financial relationships that could be construed as a potential conflict of interest.
